# Blood and adipose tissue steroid metabolomics and mRNA expression of steroidogenic enzymes in periparturient dairy cows differing in body condition

**DOI:** 10.1038/s41598-022-06014-z

**Published:** 2022-02-10

**Authors:** K. Schuh, S. Häussler, H. Sadri, C. Prehn, J. Lintelmann, J. Adamski, C. Koch, D. Frieten, M. H. Ghaffari, G. Dusel, H. Sauerwein

**Affiliations:** 1 Institute of Animal Science, Physiology Unit, University of Bonn, 53115 Bonn, Germany; 2 Department of Life Sciences and Engineering, Animal Nutrition and Hygiene Unit, University of Applied Sciences Bingen, 55411 Bingen am Rhein, Germany; 3 Department of Clinical Science, Faculty of Veterinary Medicine, University of Tabriz, 5166616471 Tabriz, Iran; 4 Helmholtz Zentrum München, German Research Center for Environmental Health, Metabolomics and Proteomics Core, 85764 Neuherberg, Germany; 5 Institute of Experimental Genetics, Helmholtz Zentrum München, German Research Center for Environmental Health, 85764 Neuherberg, Germany; 6 Department of Biochemistry, Yong Loo Lin School of Medicine, National University of Singapore, Singapore, 117597 Singapore; 7 Institute of Biochemistry, Faculty of Medicine, University of Ljubljana, 1000 Ljubljana, Slovenia; 8 Educational and Research Centre for Animal Husbandry, Hofgut Neumuehle, 67728 Muenchweiler an der Alsenz, Germany; 9 Thünen Institute of Organic Farming, 23847 Westerau, Germany

**Keywords:** Metabolism, Fat metabolism

## Abstract

In high-yielding dairy cows, the rapidly increasing milk production after parturition can result in a negative nutrient balance, since feed intake is insufficient to cover the needs for lactation. Mobilizing body reserves, mainly adipose tissue (AT), might affect steroid metabolism. We hypothesized, that cows differing in the extent of periparturient lipomobilization, will have divergent steroid profiles measured in serum and subcutaneous (sc)AT by a targeted metabolomics approach and steroidogenic enzyme profiles in scAT and liver. Fifteen weeks antepartum, 38 multiparous Holstein cows were allocated to a high (HBCS) or normal body condition (NBCS) group fed differently until week 7 antepartum to either increase (HBCS BCS: 3.8 ± 0.1 and BFT: 2.0 ± 0.1 cm; mean ± SEM) or maintain BCS (NBCS BCS: 3.0 ± 0.1 and BFT: 0.9 ± 0.1 cm). Blood samples, liver, and scAT biopsies were collected at week −7, 1, 3, and 12 relative to parturition. Greater serum concentrations of progesterone, androsterone, and aldosterone in HBCS compared to NBCS cows after parturition, might be attributed to the increased mobilization of AT. Greater glucocorticoid concentrations in scAT after parturition in NBCS cows might either influence local lipogenesis by differentiation of preadipocytes into mature adipocytes and/or inflammatory response.

## Introduction

**Figure 1:**
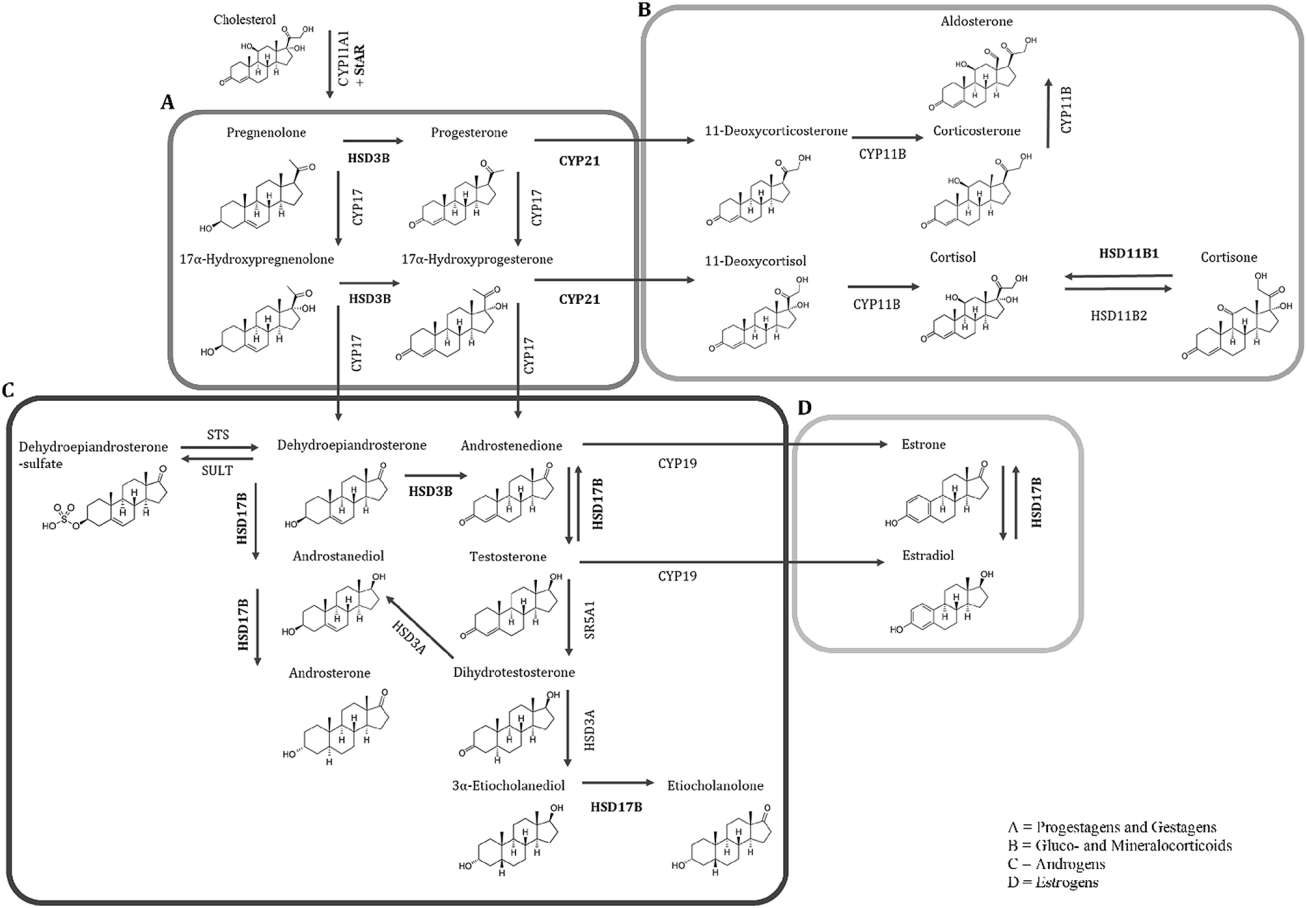
Biosynthetic pathway for steroid hormones. Biosynthetic pathway for (**A**) progestagens, (**B**) gluco- and mineralocorticoids, (**C**) androgens, and (**D**) estrogens. Enzymes involved in the steroidogenic pathway are shown next to the arrows, those enzymes being detected in the present study were highlighted in bold (the figure was adapted from Fig. 1 in MacKenzie et al.^60^. CYP11A1: cholesterol monooxygenase, CYP11B2: aldosterone synthase, CYP17: steroid-17α-hydroxylase, CYP19: aromatase, CYP21: steroid 21-hydroxylase, HSD3A: 3α-hydroxysteroid dehydrogenase, HSD3B: 3β-hydroxysteroid dehydrogenase, HSD11B1: 11ß-hydroxysteroid dehydrogenase type 1, HSD11B2: 11 ß-hydroxysteroid dehydrogenase type 2, HSD17B12: 17ß-hydroxysteroid dehydrogenase type 12, SR5A1: steroid-5α-reductase, StAR: steroidogenic acute regulatory protein, STS: steroid sulfatase, SULT: sulfotransferase.

The periparturient period in high-yielding dairy cows is associated with extensive physiological and metabolic adaptations. With the onset of lactation, the energy requirements for milk synthesis increase within a short period, during which the energy intake is commonly insufficient to meet the energy demands of the animals. Consequently, energy stores, mainly fat from adipose tissue (AT), are mobilized^1^. Adipose tissue is a highly active metabolic and endocrine organ, secreting hormones and cytokines into the circulation^2,3^. Due to their lipophilic character, steroid hormones can be stored and further metabolized by steroidogenic enzymes expressed in AT, thus modulating local steroid concentrations^3–5^. Moreover, adipocytes have the potential to synthesize steroids de novo from cholesterol and its precursors^6^. From the precursor steroids dehydroepiandrosterone (DHEA) and DHEA-sulfate (DHEA-S) steroidogenic enzymes such as 3 β-hydroxysteroid dehydrogenases (HSD3) and 17 ß-hydroxysteroid dehydrogenases (HSD17) locally synthesize androgens and/or estrogens^7,8^. The enzyme aromatase (CYP19) generates estrogens from androgens, i.e. from androstenedione and testosterone. Besides transforming estradiol to estrone, the enzyme HSD17 type 12 (HSD17B12) is involved in the elongation processes of very-long-chain fatty acids (VLCFA)^9^. Furthermore, HSD17B12 is highly expressed in organs related to fatty acid (FA) synthesis^10^. The steroidogenic acute regulatory protein (StAR) triggers cholesterol delivery to the inner mitochondrial membrane^6^. From there, the cholesterol side-chain cleavage enzyme (CYP11A1, also known as P450scc) initiates steroidogenesis by converting cholesterol to pregnenolone, acting as a precursor for all endogenous steroids^11^. The biosynthetic pathways for progestagens and gestagens, gluco- and mineralocorticoids, androgens, and estrogens are shown in Fig. 1 (main enzymes involved in steroid biosynthesis considered in the present study are highlighted within the pathways).

In humans, steroidogenic enzymes are involved in processes regulating obesity and central fat accumulation^3^. Moreover, sex steroids such as estrogens and androgens can participate in the regulation of body fat distribution and can locally influence AT function^12^. Newell-Fugate^13^ reviewed several effects of steroids, in particular of androgens and estrogens, on AT functions such as lipogenesis, lipolysis, adipocyte differentiation, insulin sensitivity as well as on adipokine secretion, mainly in monogastric species. In high-yielding dairy cows, an elevated body condition score (BCS > 3.5) before parturition is associated with increased lipolysis and ketogenesis^14^ as well as increased incidence of metabolic disorders during the transition period^1^. Throughout lipomobilization, not only FA, but also steroids can be released from AT into the circulation^15,16^, contributing to the whole body`s steroid level. So far, studies of circulating steroids in dairy cows were mainly focused on fertility and reproduction; however, the amount of steroids synthesized and metabolized in bovine AT as well as their relation with the circulating concentrations has not been investigated. Moreover, regarding the putative relevance of steroids in AT, studies addressing the transition period in context with increased lipomobilization due to different body conditions of dairy cows are lacking to our knowledge.

In this study, we used an experimental model for dairy cows investigating high versus normal body tissue mobilization during the transition from pregnancy to lactation. Based on a targeted divergence in body condition in late lactation^14^ we aimed to investigate the impact of body condition and lipomobilization on circulating and on AT specific steroid profiles from normal and over-conditioned cows around calving. We hypothesized that cows being distinct in the extent of periparturient lipomobilization, will also differ in their steroid and steroidogenic enzyme expression profiles. Thus, cows with a higher body condition around parturition will release more fat from AT and will have increased circulating steroid concentrations in peripheral blood when compared to cows with normal body condition. We aimed at characterizing the mRNA abundance of major steroidogenic enzymes in AT and liver and to compare the steroid hormone profiles in blood serum and in AT assessed by a targeted metabolomics approach. The objectives of the present study were (1) to describe the concentrations of steroid hormones, their precursors and metabolites in serum and subcutaneous AT (scAT) from late pregnancy through early lactation, (2) to assess the mRNA abundance of different key enzymes involved in steroid biosynthesis during the transition from late pregnancy to lactation in scAT and liver, and (3) to compare the steroid concentrations as well as the mRNA abundance of steroidogenic enzymes in dairy cows with high (HBCS) versus normal body condition score (NBCS) from late pregnancy through early lactation.

## Results

### Principal component analysis (PCA) and two-way heatmap clustering

**Figure 2:**
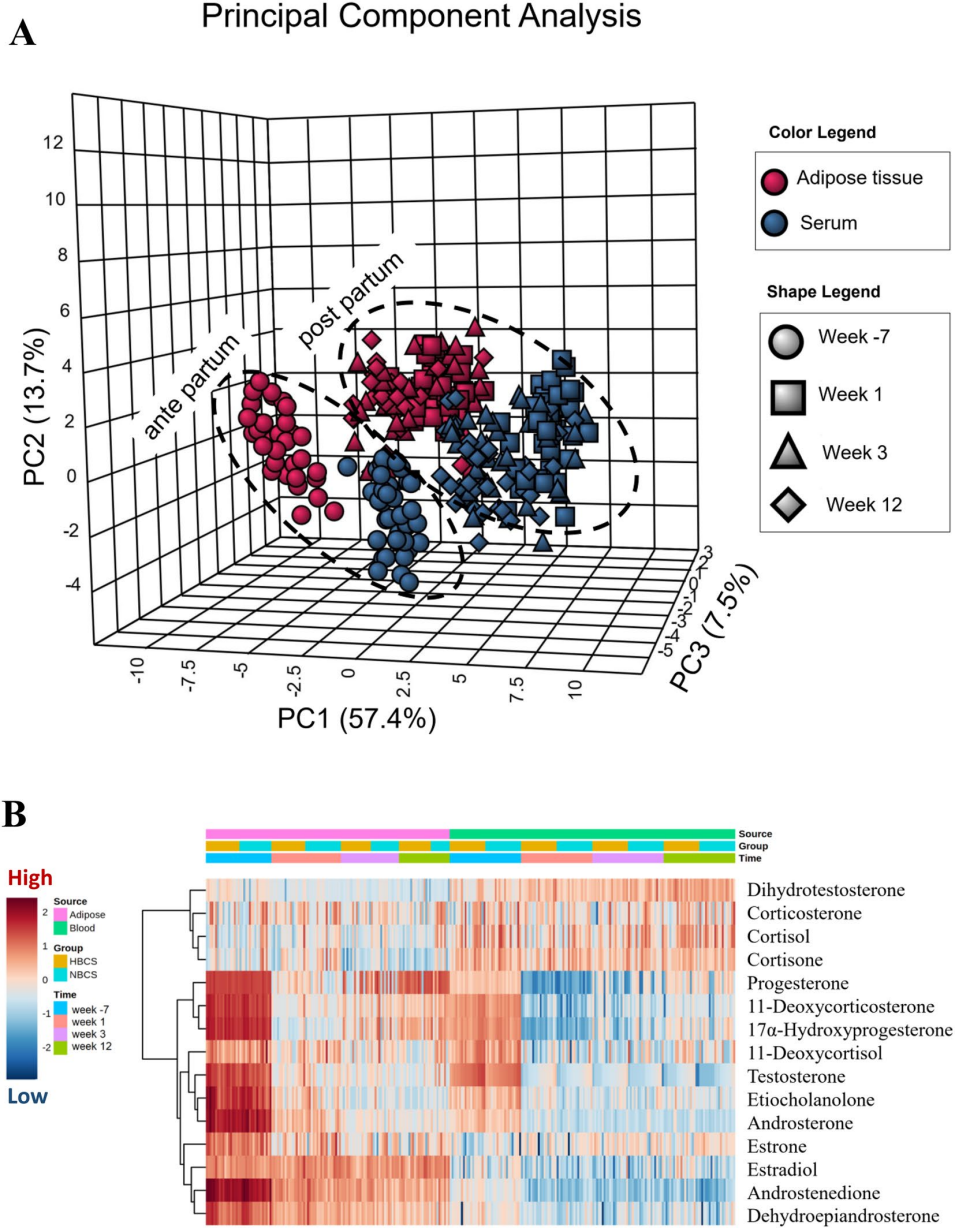
Principle component analysis and clustering of steroids. (**A**) Principal component analysis (PCA) showing the interaction of source (colored, adipose tissue and serum) and time of sampling (shape, weeks −7, 1, 3, 12 relative to parturition) for all log-transformed and pareto-scaled steroids regardless of body condition. (**B**) Clustering result for all log-transformed and pareto-scaled steroids shown as heatmap (distance measure using euclidean and clustering algorithm using ward). The color spectrum intuitively indicates the steroids abundance (mean centered and divided by the range of each variable).

Applying PCA yielded significant separations between the steroids ante partum (a.p.) and postpartum (p.p.) as well as for the steroid concentrations in serum and scAT (Fig. 2A). However, the time pattern of steroid concentrations in scAT was comparable with steroids in serum. Using hierarchical cluster analysis, we clustered the steroids (with except for pregnenolone and pregnanediol) and presented it as a heat map (Fig. 2B). The clustered heat map shows steroids in two main clusters. The first cluster contains glucocorticoids (cortisol, cortisone, and corticosterone) and the second cluster contains all other steroids (progestagens, androgens, and estrogens).

### Profiles of steroid hormones in serum

**Figure 3:**
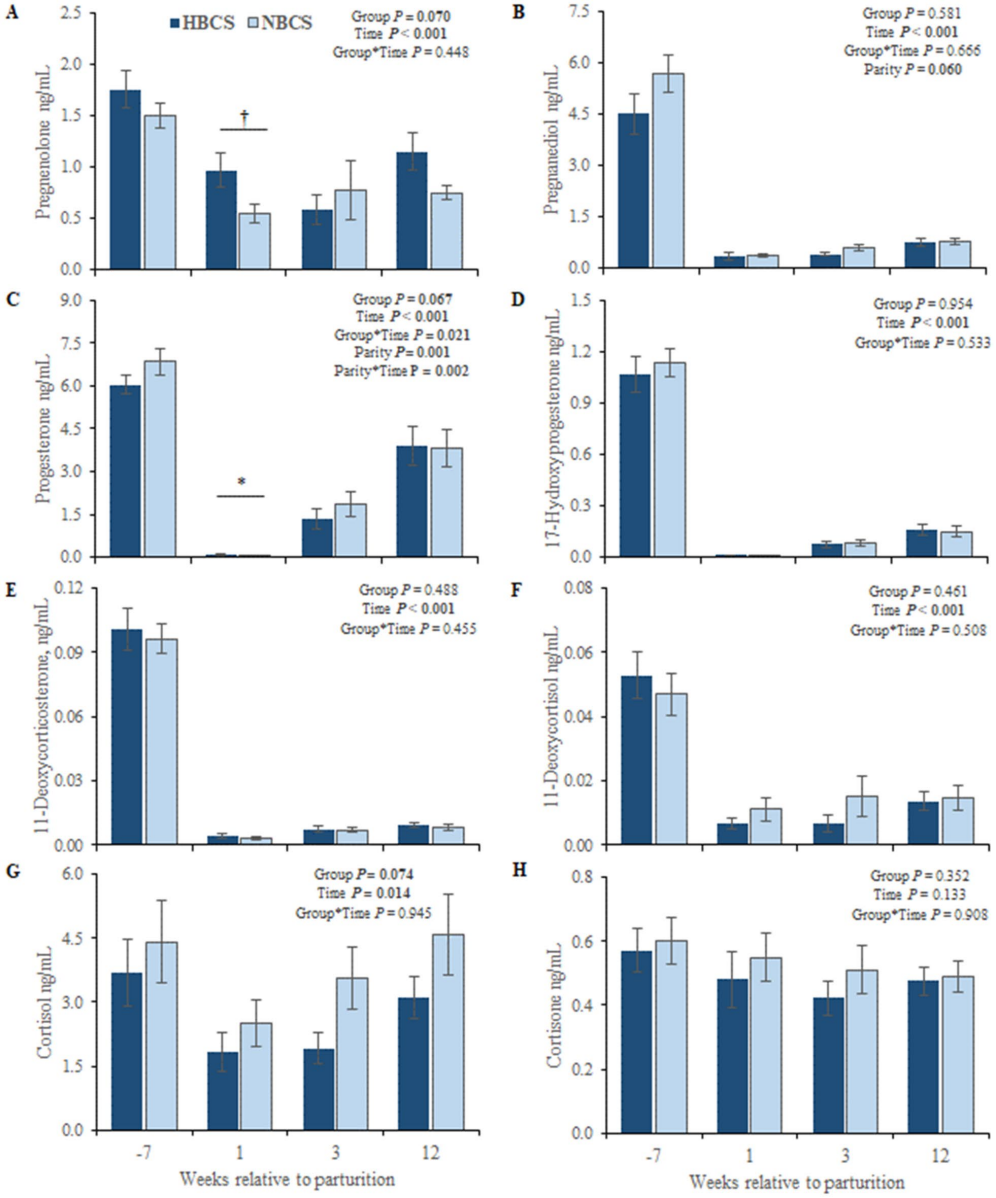

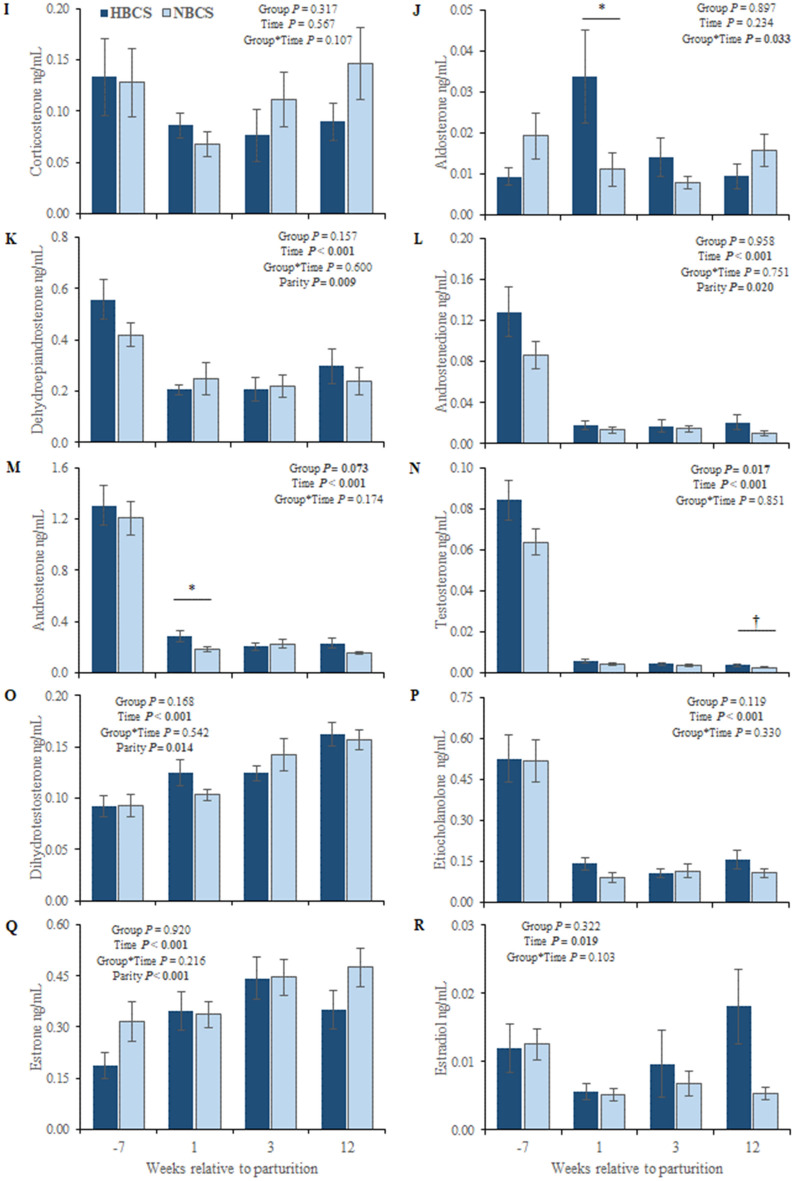
Steroid hormones in serum. Changes of the steroid concentrations (ng/mL) in serum of cows with normal versus high body condition score (NBCS, HBCS) at week 7 ante partum, as well as week 1, 3 and 12 postpartum. Data are given as means ± SEM. Asterisks (*) indicate differences (*P* ≤ 0.05) between HBCS and NBCS cows within the time points. Trends (*P* ≤ 0.10) for differences between the groups are indicated by daggers (†).

Steroid hormones measured in serum from HBCS and NBCS cows at weeks −7, 1, 3, and 12 relative to parturition are presented in Fig. 3. Overall, DHEA-S was not detectable in serum. Most blood steroids changed over time with higher concentrations before than after parturition; however, the concentrations of the mineralo- and glucocorticoids aldosterone, corticosterone, cortisone, and cortisol as well as the estrogens, estrone, and estradiol, followed different patterns. Group differences were observed at week 1 p.p. for progesterone, aldosterone, and androsterone with higher concentrations (1.1-fold to threefold, *P* < 0.05) in HBCS than in NBCS cows. For progesterone, an interaction between group and time was observed (*P* = 0.02). Regarding parity, cows from parity class 2 (≥ 2nd and < 4th parity) had higher concentrations of progesterone, estrone (E1), dihydrotestosterone (DHT), and DHEA with nearly twice the concentrations compared to cows in parity class 3 (> 4th parity; *P* < 0.05). In contrast, cows from parity class 3 had 2.3-fold higher androstenedione concentrations compared to cows from parity class 2. Moreover, circulating mineralo- and glucocorticoids were not affected by parity.

**Figure 4:**
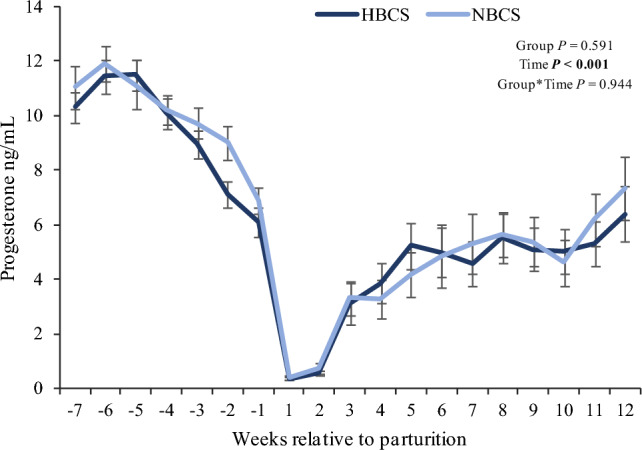
Progesterone concentrations in serum. Changes in serum progesterone concentration (ng/mL) in the serum of cows with normal versus high body condition score (NBCS, HBCS) from week 7 ante partum to week 12 postpartum measured by ELISA. The vertical dashed line indicates parturition. Data are given as means ± SEM. Significant value: *P* ≤ 0.05.

The progesterone concentrations in serum measured by ELISA were neither affected by group nor by parity (Fig. 4). The values measured by ELISA were consistently higher (around 68%) throughout the experimental period when compared to progesterone values measured by ultra-high-performance liquid chromatography-tandem mass spectrometry (LC–MS/MS). Moreover, progesterone values measured by ELISA and mass spectrometry (MS) in the 4-time points from which MS data were available, were strongly correlated (r = 0.90; *P* < 0.001).

### Profiles of steroid hormones in scAT

**Figure 5:**
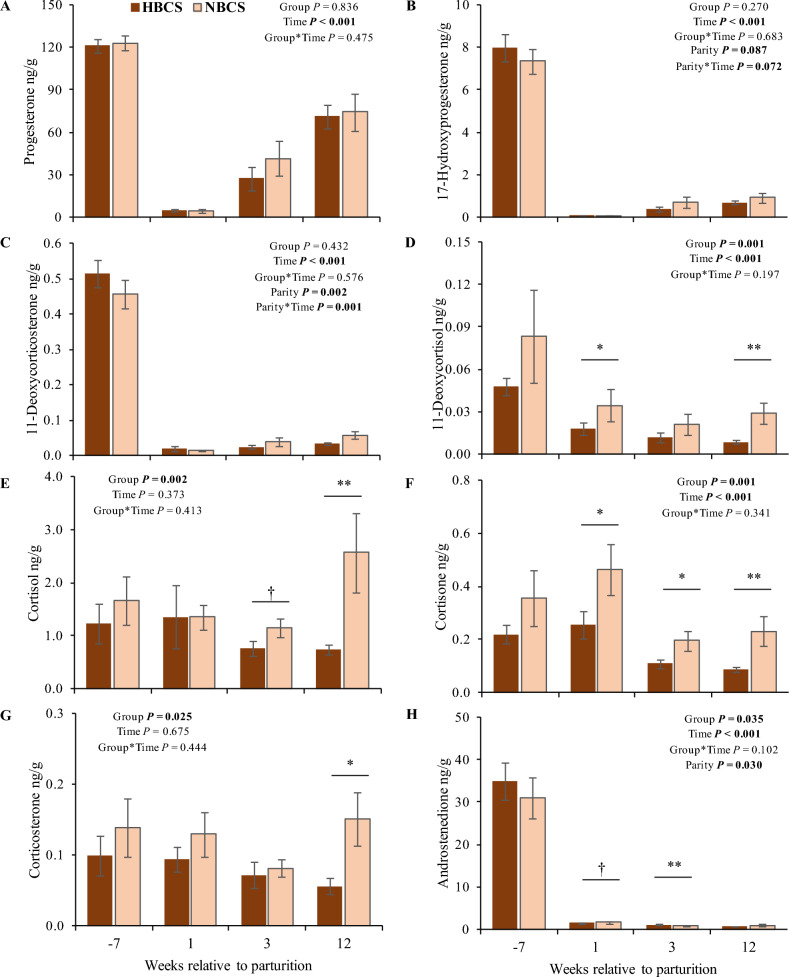

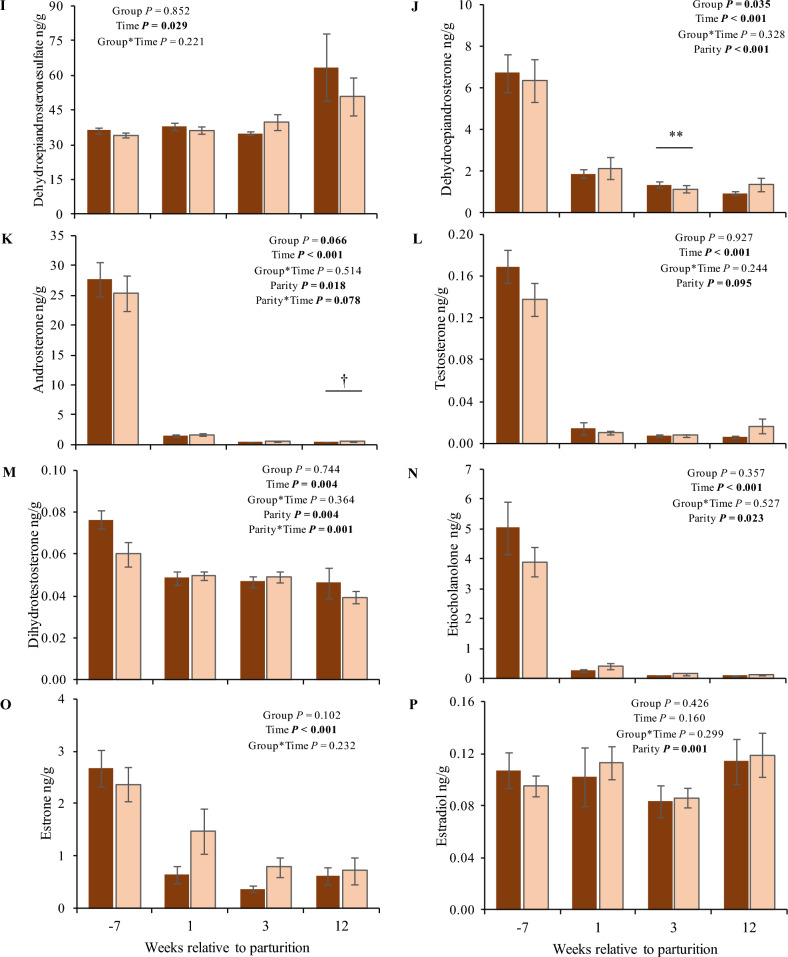
Steroid hormones in subcutaneous adipose tissue. Changes of the steroid concentrations (ng/g) in subcutaneous adipose tissue of cows with normal versus high body condition score (NBCS, HBCS) at week 7 ante partum, as well as week 1, 3 and 12 postpartum. Data are given as means ± SEM. Asterisks indicate differences (*P ≤ 0.05; **P ≤ 0.01) between HBCS and NBCS cows within the time points. Trends (*P* ≤ 0.10) for differences between the groups are indicated by daggers (†).

The steroid hormones measured in scAT are presented in Fig. 5. In general, the most abundant steroid in scAT was progesterone, whereas aldosterone was not detectable in scAT (aldosterone concentrations were below the limit of detection (LOD)). Group differences in scAT (observed for 11-deoxycortisol (11-DOC), corticosterone, cortisol, cortisone, androstenedione, and DHEA) were limited to the phase after parturition. For the mineralo- and glucocorticoids, concentrations were higher (1.9- to 3.5-fold, *P* < 0.05) in NBCS than in HBCS cows, whereas DHEA and androstenedione were higher (1.2- and 1.5-fold, respectively, *P* < 0.005) in HBCS compared to NBCS cows. Irrespective of time and group, cows from parity class 2 (≥ 2nd and < 4th parity) had up to 1.8-fold higher concentrations of estradiol (E2), androstenedione, and DHEA in scAT compared to cows from parity class 3 (> 4th parity).

### mRNA abundance of steroidogenic enzymes in liver and scAT

**Figure 6:**
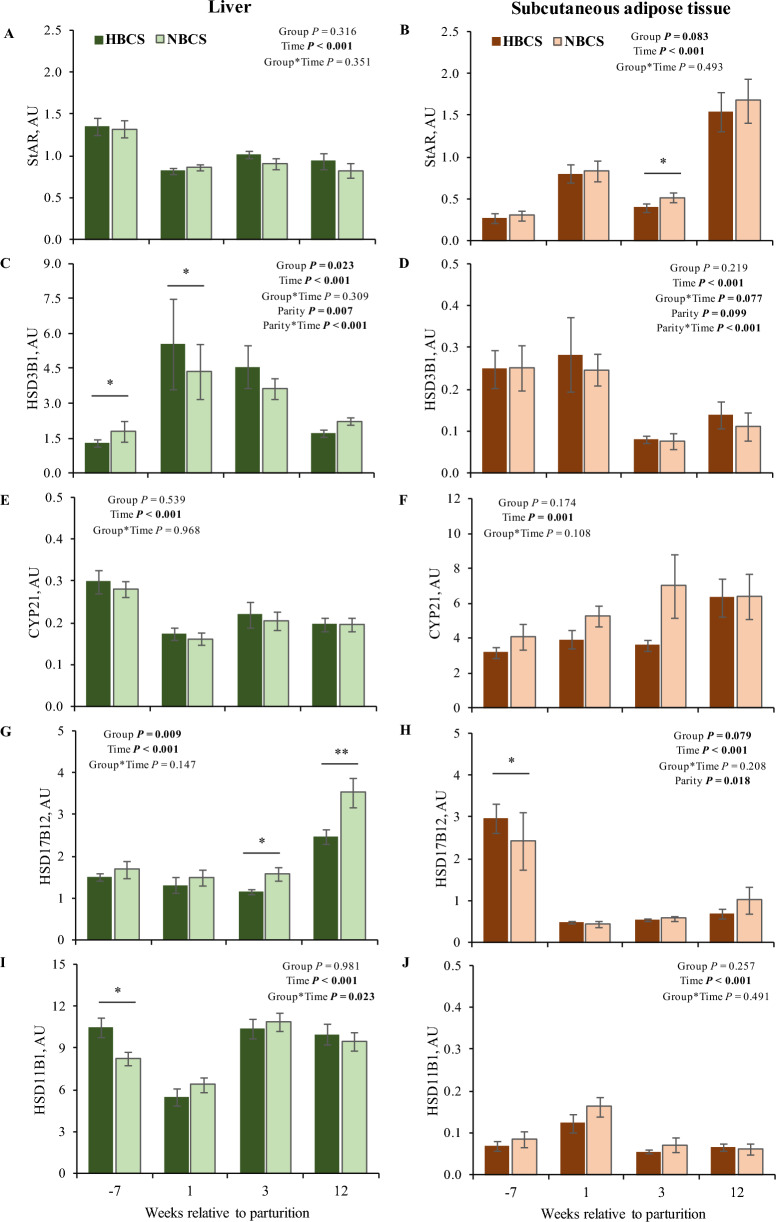
mRNA abundance of steroidogenic enzymes in liver and subcutaneous adipose tissue. mRNA abundance of five steroidogenic enzymes [steroidogenic acute regulatory protein (*StAR*), 3 β-Hydroxysteroid dehydrogenase type 1 (*HSD3B1*), steroid 21-hydroxylase (*CYP21*), 17 ß-hydroxysteroid dehydrogenase type 12 (*HSD17B12*), and 11 ß-hydroxysteroid dehydrogenase type 1 (*HSD11B1*)], in the liver and in subcutaneous adipose tissue of cows with normal versus high body condition score (NBCS, HBCS) at wk 7 ante partum, as well as wk 1, 3 and 12 postpartum (time = weeks around parturition). Asterisks indicate differences (*P ≤ 0.05; **P ≤ 0.01) between HBCS and NBCS cows within the time points. Trends *(P* ≤ 0.10) for differences between the groups are indicated by daggers (†). AU: arbitrary unit.

The abundance of steroidogenic enzymes mRNA in liver and scAT are presented in Fig. 6. Comparing the tissues, the mRNA abundance of *HSD3B1 (HSD3 type 1)* and *HSD11B1 (11 ß-hydroxysteroid dehydrogenase type 1)* was higher (*P* < 0.001) in liver compared to scAT at week 3 p.p. In contrast, the *steroid 21-hydroxylase* (*CYP21*) mRNA abundance was higher (*P* < 0.001) in scAT than in liver at all time points (Fig. 6E and F). Before parturition, higher *HSD17B12* mRNA abundance was observed in scAT compared to liver (*P* = 0.008); however, after calving the mRNA abundance of *HSD17B12* was higher in liver (*P* < 0.001, Fig. 6G and H). Regarding group differences, the mRNA abundance of *HSD17B12* in liver was higher (*P* < 0.05) in NBCS cows compared to HBCS cows at weeks 3 and 12 p.p. Moreover, the hepatic mRNA abundance of *HSD11B1* was higher (*P* = 0.010) at week 7 a.p. in HBCS compared to NBCS cows (Fig. 6I). In scAT, group differences were limited to *StAR* and *HSD17B12*, being higher at week 3 p.p. (*StAR; P* = 0.035; Fig. 6B) in NBCS compared to HBCS cows and before parturition (*HSD17B12; P* = 0.032; Fig. 6H) in HBCS compared to NBCS cows.

Parity affected *HSD3B1* mRNA abundance in liver, with higher abundance in cows from parity class 3 (> 4th parity) compared to class 2 (≥ 2nd and < 4th parity). In addition, the mRNA abundance of *HSD3B1* in the scAT was higher in cows from parity class 3 compared to cows from class 2 at weeks 7 a.p. and 12 p.p. (*P* ≤ 0.05), respectively. The mRNA abundance of *HSD17B12* in scAT across all time-points was higher (*P* = 0.02) in cows with parity > 4 than in cows from class 2 (≥ 2nd and < 4th parity). For *CYP21* mRNA abundance in liver, neither group nor parity of the cows affected the enzyme expression (Fig. 6E). Moreover, parity did not affect *HSD11B1* mRNA abundance in both liver and scAT.

### Relationships between the steroid hormone concentrations in serum, in scAT, and the mRNA abundance of steroidogenic enzymes in scAT

**Figure 7:**
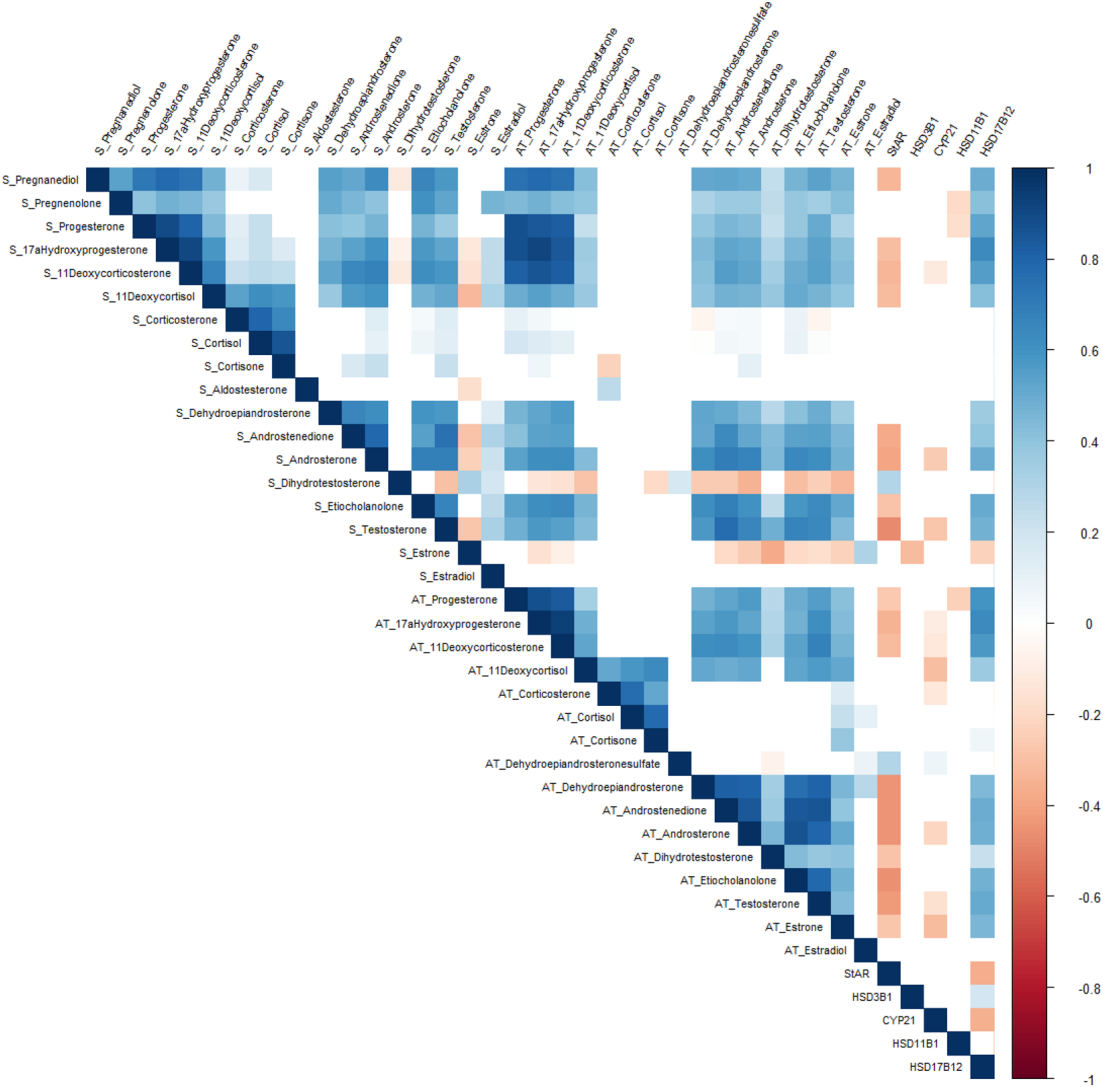
Relation between steroid hormones in serum and subcutaneous adipose tissue as well as steroidogenic enzyme mRNA. Spearman correlation plots of the steroids assessed in serum (S) and in subcutaneous adipose tissue (scAT) as well as with the mRNA abundance of steroidogenic enzymes in scAT. The correlations are based on results of overall time points and group independence. Squares are colored based on correlation coefficients (−1 to + 1) with *P* < 0.05.

The relationships between steroid concentrations in serum and scAT as well as the associations between steroids and mRNA abundance of steroidogenic enzymes in scAT regardless of grouping and time are presented in Fig. 7. Very strong correlations (r > 0.9) were observed for 11-deoxycorticosterone (11-DOCSt) and 17α-hydroxyprogesterone (17-OHP) in serum (r = 0.905; *P* < 0.001), 17-OHP in serum and 17-OHP in scAT (r = 0.916; *P* < 0.001) as well as for 17-OHP and 11-DOCSt in scAT (r = 0.931; *P* < 0.001).

### Relationships between the mRNA abundance of steroidogenic enzymes in liver and the concentration of steroids in the circulation

Regardless of grouping and time, the mRNA abundance of enzymes *HSD11B1*, *HSD17B12*, and *StAR* in the liver were very weakly associated with blood steroids (r < 0.40; *P* < 0.001). However, the mRNA abundance of *CYP21* was moderately related to pregnanediol (r = 0.45), pregnenolone (r = 0.44), testosterone (r = 0.47), 11-DOCSt (r = 0.47), androstenedione (r = 0.46), androsterone (r = 0.40), etiocholanolone (r = 0.41), and 17-OHP (r = 0.45) (all *P* < 0.001). Furthermore, the mRNA abundance of *HSD3B* in liver was negatively associated (r < −0.40) with blood steroids, i.e. 11-DOCst, 11-DOC, 17-OHP, androstenedione, androsterone, cortisol, cortisone, DHEA, etiocholanolone, pregnanediol, pregnenolone, and testosterone; except for progesterone, a moderate negative relation was observed with hepatic *HSD3B mRNA* (r = −0.42; *P* < 0.001).

## Discussion

Steroid hormones are involved in various physiological processes during gestation and lactation. Due to their lipophilic character, steroids can be taken up from circulation and accumulated in AT^5,6^. In humans, steroids can be locally synthesized de novo or at least be metabolized by steroidogenic enzymes from steroid precursors^11^. With this background, we profiled the steroid concentrations in serum and scAT of periparturient dairy cows using a targeted metabolomics approach. The cows differed in their body condition before parturition, which maintained until calving; the difference in body condition was augmented by feeding a more energy-dense diet to the HBCS cows from week 15 a.p. until dry-off (7 a.p.). In particular, HBCS cows with higher BCS and BFT throughout the experimental period, lost more than twice as much BFT during early lactation when compared to NBCS cows^14^. Therefore, we hypothesized that cows with a higher body condition around parturition, releasing more fat from AT after calving, will have increased circulating steroid concentrations when compared to cows with normal body condition. Greater lipomobilization in early lactation substantiated by greater serum FA concentrations^14^ might lead to an increased release of steroids from AT, and thus enhanced circulating steroid concentrations, in particular in HBCS cows. Cows were fed differently from wk 15 to wk 7 before the anticipated calving date to reach the targeted BCS and BFT until dry-off. Thus, it is likely that the observed responses in this experiment on wk −7 might have been affected by differential feeding during late lactation. Moreover, a potential effect of differential feeding during the late lactation on AT constitution is also likely, but was not evaluated in this study.

Using gas chromatography, Bélanger et al*.* observed regional differences in human AT steroid levels, being more concentrated in omental versus scAT^17^. In addition, investigating steroid concentrations in different human AT depots (i.e. breast and abdominal AT), Szymczak et al*.* suggested local factors being involved in steroid uptake, storage, act, and metabolism^18^. Therefore, we would expect differences in steroid biosynthesis and metabolism between visceral and scAT depots in bovine. Nevertheless, besides regional differences, steroid concentrations in plasma were strongly correlated with steroid values both in omental and scAT in humans^17^. Given that biopsies from different visceral AT depots cannot routinely be sampled for tissue analyses in the alive cow, since the invasive overall sampling of AT could impair the metabolism of cows during the already challenging transition period. Therefore, we focused on scAT in the present study, albeit differences in the functional activities of visceral and scAT have been shown for dairy cows^19,20^.

In the course of gestation, most steroid concentrations increase and peak near the end of term in maternal serum, whereas the steroid concentrations after parturition mostly decrease. Thus, concentrations below the LLOQ were generally found after parturition in this study. Herein, more than 50% of serum steroids measured were below the LLOQ. Moreover, concerning 11-DOCSt, testosterone, androstenedione, and 11-DOC, 75–53% of the concentrations were below the LLOQ. The steroid concentrations in scAT and serum clearly differed with respect to the time before and after parturition in the current study. Furthermore, from the PCA analysis, glucocorticoids (except 11-DOC and 11-DOCst) and DHT showed distinct changes in concentration within time, source (scAT and serum), and group (NBCS and HBCS) compared to most of the sex steroids, pointing to the different function of these hormones in terms of reproduction and metabolism.

Increased circulating progesterone in cows with high versus normal body weight loss is attributed to fat mobilization after parturition and the release of progesterone stored in AT^15,16^. In the present study, greater lipomobilization in HBCS cows was accompanied by higher serum concentrations for progesterone, aldosterone, and androsterone at week 1 p.p. when compared to NBCS cows. Moreover, the trend for elevated circulating pregnenolone and the greater progesterone concentrations in the HBCS cows at week 1 p.p. might originate from increased AT mobilization in these cows. In dairy cows, the corpus luteum is the major contributor to the elevated peripheral progesterone levels during pregnancy^21^. Weekly measured progesterone concentrations in the present study followed the well-known changes of the hormone concentrations, appearing in context with parturition, i.e. progesterone decreases prior to the time of parturition and increases thereafter. Consistently higher values measured by ELISA throughout the whole experimental period compared to LC–MS/MS values, raise the problem that using different methods for quantification of the same hormone is difficult for direct comparison. However, using LC–MS/MS for multiple steroid quantification herein included also progesterone concentrations, being highly correlated to progesterone concentrations measured by ELISA.

As in humans hyperaldosteronism was associated with metabolic syndrome, obesity, and insulin resistance^22^, higher serum aldosterone concentrations at week 1 p.p. in HBCS than in NBCS cows, together with increased insulin concentrations in the periparturient period^14^, might reflect reduced insulin sensitivity in HBCS cows. Comparing the present results with concentrations of FA published earlier^14^ (see Supplemental Fig. 2) circulating aldosterone was only weakly associated with FA in week 1 p.p. (r = 0.40; *P* = 0.03, both groups). The relevance of androgens in female reproduction is primarily focused on their role as estrogen precursors. However, androgens per se might regulate key processes during pregnancy and parturition, e.g., androgens are believed to be critical for cervical remodeling at term and in myometrial relaxation in humans. In particular, androsterone might play a role in myometrial contractions^23^. In humans, hyperandrogenism is a key feature of polycystic ovary syndrome, with increased androgen concentrations being related to insulin resistance^24^. As mentioned for aldosterone, higher androgen concentrations, i.e. androstenedione and DHEA in scAT as well as androsterone in serum, from HBCS cows in the present study might reflect reduced insulin sensitivity in these cows. Besides interference with insulin signaling, androgens may also trigger lipolysis and thus increase FA in circulation^24^. In addition to steroids, we assessed the mRNA abundance of five key enzymes involved in steroid biosynthesis in the present study. Besides in liver, steroids can be metabolized by steroidogenic enzymes in scAT^25^ and thus modulate steroid concentrations^11^. Endogenous steroids originate from their common precursor cholesterol. Together with mitochondrial outer-membrane proteins such as the translocator protein (TSPO)^26^, the enzyme StAR triggers the delivery of cholesterol into the inner mitochondrial membrane (IMM), while CYP11A1 can initiate steroidogenesis through a side-chain cleavage reaction converting cholesterol to pregnenolone^6^. In mammals, pregnenolone is the main steroid synthesized from cholesterol, initiated by StAR and CYP11A1^6,11^ serving as a precursor for other steroids. Pregnenolone, taken up from the circulation, can be converted to 17-OHP by CYP17A1^11^. In the current study, *CYP11A1* mRNA was not detectable. Studies in murine adipocytes indicate that CYP11A1 may play only a minor metabolic role in AT^6^. However, the *StAR* mRNA was measured in scAT in our study and peaked at week 12 p.p. The higher *StAR* mRNA abundance in scAT from NBCS compared to HBCS in early lactation indicates an increased capacity of cholesterol uptake into the IMM at this time point. Cholesterol reaching the IMM can alternatively be metabolized to oxysterols instead of pregnenolone. Oxysterol 27-hydroxycholesterol is one of the major de novo adipocyte products synthesized from cholesterol by the mitochondrial enzyme CYP27A1^6^. The de novo synthesis of oxysterols in adipocytes was suggested to protect adipocytes against intracellular cholesterol overload and the formation of new fat cells, thus controlling the number of adipocytes upon overnutrition^6^. Increased abundance of *StAR* mRNA in scAT of NBCS cows in the present study may therefore reflect oxysterol synthesis.

Regarding the steroid concentrations in scAT, higher DHEA and androstenedione concentrations in HBCS cows p.p. may point to either an increased lipid accumulation or to a higher local metabolism of these steroids. The enzyme CYP17A1 modulates the transformation of pregnenolone to DHEA; however, in the present study, *CYP17A1* mRNA abundance was not detectable in scAT with the protocol used herein. Considering the enzyme *HSD3B1*, increased hepatic mRNA abundance in the week after parturition was accompanied by increased androstenedione concentrations in scAT in HBCS cows p.p., thus the enzyme might contribute to peripheral conversion of androstenedione from DHEA. Both androgen precursors, DHEA and DHEA-S, were present in scAT of cows in our study, but DHEA-S was below the LLOQ in all serum samples measured herein. We cannot explain the absence of DHEA-S in bovine serum samples in this study; however, earlier studies have also observed lower DHEA-S than DHEA concentrations in circulation, suggesting a limited contribution of DHEA-S as an androgen reservoir in cows^27^. In the current study, two steroidogenic enzymes, *HSD17B12* and *HSD3B1*, involved in androgen biosynthesis were expressed in bovine scAT and liver. In humans and rodents, androgens inhibit adipogenesis and promote lipid mobilization via androgen receptors in AT^25,28^. Also, androgens can enhance the lipolytic capacity of preadipocytes by increasing the number of ß-adrenergic receptors and the activity of the enzyme adenylate cyclase^25,28^. Higher androgen concentrations in scAT from HBCS may thus prevent adipogenesis in early lactation when these cows were still mobilizing body reserves^14^ (Supplemental Fig. 1). Moreover, the higher mRNA abundance of *HSD3B1* in liver compared to scAT may point to hepatic synthesis of progesterone, albeit the main site of progesterone synthesis in dairy cows to maintain pregnancy is the corpus luteum^21^. Locally converted progesterone could have an anabolic role in bovine liver, since administration of progesterone increased the rate of hepatic lipogenesis in rats *in vivo*^29^.

The interconversion of active 17-ß-hydroxy- and inactive 17-keto-steroids is catalyzed by HSD17-forms and plays an essential role in the last steps of androgen and estrogen biosynthesis. In our study, we investigated the mRNA abundance of *HSD17B12* catalyzing the synthesis of E2 from E1^9^. Moreover, HSD17B12 is involved in the elongation process of VLCFA and is highly expressed in organs related to lipid metabolism, e.g., liver, kidney, heart, and skeletal muscle^10,30^. Depending on the concentration, *HSD17B12* could catalyze both, the elongation of FA, as well as the transformation of sex steroids^31^. Higher *HSD17B12* mRNA abundance in HBCS cows at week 7 a.p. might point to a role of the enzyme in lipid metabolism. Furthermore, higher hepatic abundance of *HSD17B12* mRNA in NBCS than HBCS cows after parturition may contribute to the same mechanism, since the NBCS cows returned earlier to a positive energy balance^14^, which is likely associated with lipogenic processes. The association between *HSD17B12*, E1, and etiocholanolone, suggests a role of *HSD17B12* in converting DHT to etiocholanolone rather than E1 to E2. Furthermore, higher E1 concentrations in scAT compared to serum may be due to greater E1 storage, increased local E1 synthesis in scAT, or a combination of both. Since E1 was increased relative to E2 in our study, rather the oxidative pathway than the reductive pathway catalyzed by HSD17 seems to be relevant. However, the gene expression of estrogenic *HSD17* enzymes in AT was reported to be lower than those of the androgenic ones; thus, androgen biosynthesis might be more relevant in AT than estrogen biosynthesis^32^. Also, the higher E1 concentrations in the scAT of our study might be due to local estrogen synthesis via steroid sulfatase enzyme (STS) and CYP19 aromatization of androstenedione^33^. However, aromatase CYP19 was not investigated in the current study. In bovine maternal circulation, E1-S is the major estrogen, indicating foetoplacental function and placental viability^34^. E1-S serves as a hormone reservoir in the circulation; in general, sulfonated steroids often exceed the concentrations of free (unconjugated) steroids in the circulation and different tissues, as also shown for DHEA-S in scAT in our study. The E1 concentrations in scAT of the cows in our study may be due to the action of STS as well as to uptake and conversion of E1-S.

Being generally known as stress hormones, glucocorticoids are mainly produced in the adrenal glands to stimulate the release of energy substrates from energy stores for use as fuel during the stress response^35^. Moreover, glucocorticoids have immune-suppressive and anti-inflammatory effects on several organs, including AT^36,37^. In vivo and in vitro studies in humans and rodents have shown that glucocorticoids also regulate lipid metabolism by stimulating lipolysis^38^, diminishing preadipocyte proliferation^39^, but also by encouraging adipogenesis through stimulation of differentiation from pre- to mature adipocyte^40^. In the present study, the circulating concentrations of the active glucocorticoid cortisol were within the range reported for dairy cows during the periparturient period^41^. Given that cows in a positive energy balance have no need to mobilize energy stores, the higher cortisol concentrations in scAT from NBCS cows might rather result from an accumulation of circulating cortisol, than from local synthesis. Using a proteomics analysis on plasma samples from a subset of the cows studied herein, we found that the most enriched pathways were those involved in the acute inflammatory response and regulation of humoral immune response^42^. Although glucocorticoids are not synthesized de novo in AT, they can be activated locally by HSD11B1^40^. In dairy cows, the enzyme activity of HSD11B1 has been measured in different AT depots^43^. Located exclusively in mature, differentiated adipocytes, an anti-inflammatory rather than a differentiating effect of cortisol was suggested in bovine AT^43^. Increased mRNA abundance of *HSD11B1* in scAT at week 1 p.p measured herein, might support the inflammatory role of cortisol in bovine AT, as demonstrated around parturition in bovine AT^44^. The liver mainly activates glucocorticoids; therefore, higher hepatic mRNA abundance of *HSD11B1* compared to scAT was not surprising. At week 7 a.p., the hepatic *HSD11B1* abundance in HBCS cows was moderately related to circulating beta-hydroxybutyrate at week 7 a.p. (r = 0.50; *P* < *0.05*), indicating a role in lipid metabolism and ketogenesis^40^. The formation of mineralo- and glucocorticoids is triggered by the enzyme CYP21 from the precursor steroids progesterone and 17-OHP. Herein, the higher mRNA abundance of *CYP21* in scAT than in liver may suggest that mineralo- and glucocorticoids are rather converted in scAT than in liver in cattle.

In the present study, we also aimed at investigating the effect of parity on the steroid profiles in serum and in scAT as well as on the mRNA abundance of steroidogenic enzymes. Both, endocrine and metabolic parameters are affected by parity during the periparturient period in dairy cows^45^. Higher steroid concentrations in serum and in scAT from cows in parity class 2 (≥ 2nd and < 4th parity) compared to cows with parity > 4 occurred more frequently right after calving when the physiological adaptations to lactation including the mobilization of fat reserves proceed^46^ and estrous cycles are resumed^8^. In vitro experiments revealed, that progesterone secreted from corpus luteum, as well as the mRNA expression of *StAR* and *HSD3B*1, were decreased in aged cows (mean age: 15.7 years) compared with younger cows (2.9 years) during the early luteal phase^47^. In the present study, cows with parity > 4 had more frequently higher mRNA abundances of steroidogenic enzymes in both liver and scAT compared with cows of ≥ 2nd parity and < 4th parity, suggesting an aging effect on luteal function^47^ as well as on steroidogenic enzyme expression in cattle.

## Conclusion

The results of the present study indicated that not only steroid hormones but also steroidogenic enzymes in liver and scAT were altered during the periparturient period of cows. Increased lipolysis in cows after parturition was related to higher circulating concentrations of androgens and progestagens, which might reflect the steroid release from AT into the circulation due to fat mobilization in early lactation. Moreover, the age of the cows not only affected the steroid concentration in serum and AT, but also the mRNA abundance of steroidogenic enzymes in a local manner, depending on the time relative to parturition, which requires further investigation.

## Materials and methods

The animal experiment was performed at the experimental station of the Educational and Research Centre for Animal Husbandry, Hofgut Neumuehle, Muenchweiler a. d. Alsenz, Germany. The experimental procedures performed in this study were in accordance with the European Union Guidelines concerning the protection of experimental animals, with approval by the local authority for animal welfare affairs (Landesuntersuchungsamt Rheinland-Pfalz, Koblenz, Germany [G 14-20-071]). The study is reported according to the ARRIVE guidelines. Cows were part of a trial aiming to establish an experimental model for dairy cows of high versus normal body mobilization during the transition from pregnancy to lactation. Details of the experimental design were described previously^14^. In brief, 38 multiparous German Holstein dairy cows (average parity: 2.9 ± 0.3) were allocated 15 weeks before their expected calving date to either a high BCS (HBCS, n = 19) or a normal BCS (NBCS, n = 19) group. The BCS was estimated using a scale of 1 (thin) to 5 (obese) with a quarter-point system^48^; backfat thickness (BFT) was recorded in the sacral region using ultra-sonography (AGROSCAN L, ALR 500, 5 MHz, linear-array transducer; Echo Control Medical, Angoulême, France). Both, BCS and BFT were continuously monitored every 2 weeks (from week 15 a.p. until week 12 p.p.). Changes of BCS and BFT from week 7 a.p. to week 13 p.p. in NBCS cows and HBCS cows were already documented^14^ and are shown together with BW changes as well as their time-dependent variations in Supplemental Fig. 1 and Supplemental Table 2. From week 15 until milking was stopped (“drying off”) at week 7 a.p., the HBCS and NBCS cows were assigned to two feeding groups to accentuate the differences in body condition (NBCS cows: BCS < 3.5, BFT < 1.2 cm; HBCS cows: BCS > 3.75, BFT > 1.4 cm). In addition, comparable milk yields were considered for preselection of cows (NBCS: 10,361 ± 302 kg; HBCS: 10,315 ± 437 kg). The NBCS animals were fed a less energy-dense ration [6.8 NE_L_ (MJ/kg of dry matter (DM))] than the HBCS animals [7.2 NE_L_ (MJ/kg of DM)] from week 15 a.p. until week 7 a.p. At dry off, the groups had the targeted difference in BCS and BFT: HBCS with 3.8 ± 0.1 (min/max range: 3.0 to 4.5; IQR: 0.7) and 2.0 ± 0.1 cm (min/max range: 1.3 to 2.9 cm; IQR: 0.8), respectively, and NBCS with 3.0 ± 0.1 (min/max range: 2.75 to 3.25; IQR: 0.3) and 0.9 ± 0.1 cm (min/max range: 0.4 to 1.4 cm; IQR: 0.4), respectively. During the subsequent dry period, and the lactation thereafter, both groups received the same diets. The cows obtained all diets for ad libitum intake as TMR consisting of 74% roughage and 26% concentrate in the low-energy ration and 63% roughage and 3% concentrate in the high-energy ration. A detailed description of the ingredients as well as the composition of the diets is given in Supplemental Table S1. The calculations of energy contents of the diets were performed according to the recommendations for lactating cows of the German Society of Nutrition Physiology^49^. The differences in body condition were largely maintained until calving, during lactation BCS declined in both groups, with greater BCS losses in HBCS cows than in NBCS sows (see Supplemental Fig. 1 and Supplemental Table 2).

### Sampling

Blood samples for steroid metabolomic analyses were collected after the morning milking but before feeding, on weeks −7, 1, 3, and 12 relative to parturition from the *V. caudalis mediana* as previously described^14^. Serum was obtained from blood after clotting and subsequent centrifugation (10 min, 2,000×*g* at 4 °C) and stored at −80 °C until analysis. Progesterone was measured in serum samples obtained weekly from weeks −7 until + 12 relative to parturition using an in-house developed ELISA^50^; the inter- and intra-assay coefficient of variations were 7.9% and 6.7%, respectively. Moreover, biopsies from liver as well as scAT from the tailhead region were collected from both groups on weeks −7, 1, 3, and 12 relative to parturition as described recently^51^. In brief, tissue biopsies were collected under local anesthesia (Procaine hydrochloride, 20 mg/mL, Richter Pharma AG, Wels, Austria) and while the cows were sedated (xylazine i.v., 20 mg/mL, 0.1 mL/100 kg of BW; CP-Pharma Handels GmbH, Burgdorf, Germany) and fixed in a headlock. Liver biopsies were obtained by liver puncture at the 11th and 12th intercostal space using a 14-gauge biopsy needle (Dispomed Witt oHG, Gelnhausen, Germany). For the scAT biopsies, a 1 cm skin incision was made in the tailhead region and scAT from the underlying fat layer was collected. All tissue samples were rinsed with sterile 0.9% NaCl solution, immediately snap-frozen in liquid nitrogen, and stored at −80 °C until analysis.

### Quantification of steroid hormones in serum samples

The following 19 steroids were quantified in serum (250 µL) using an extended version of the Absolute*IDQ*™ Stero17 assay (Biocrates Life Sciences AG, Innsbruck, Austria) and LC–ESI–MS/MS^52^: aldosterone, androstenedione, androsterone, corticosterone, cortisol, cortisone, 11-DOCSt, 11-DOC, DHEA, DHEA-S, DHT, E1, E2, etiocholanolone, 17-OHP, progesterone, testosterone, pregnenolone, and pregnanediol (the last two steroids were assessed semi-quantitatively). Mass spectrometric analyses were performed on a QTRAP 5500 triple quadrupole system (Sciex Deutschland GmbH, Darmstadt, Germany) equipped with a 1260 Series HPLC (Agilent Technologies Deutschland GmbH, Böblingen, Germany) and a HTC PAL autosampler (CTC Analytics, Zwingen, Switzerland) controlled by the software Analyst 1.6.2. Compound identification and quantification were based on scheduled multiple reaction monitoring measurements (sMRM). Sample preparation and LC–MS/MS measurements were performed as described by the manufacturer´s protocol (UM-STERO17, Biocrates AG). Samples were handled using a Hamilton Microlab STAR™ robot (Hamilton Bonaduz AG, Bonaduz, Switzerland) and a Waters Positive Pressure-96 Processor (Waters GmbH, Eschborn, Germany). Until analysis, all samples were stored at −80 °C. A detailed method description has been published^53^.

In brief, ultrapure water (400 µL), internal standard mix (20 µL), blank (250 µL), calibration standards, quality control samples, or serum samples were mixed in a 96 deep well plate. The SPE (solid phase extraction) plate of the kit was conditioned successively with dichloromethane (1 mL), followed by acetonitrile (1 mL), methanol (1 mL), and ultrapure water (1 mL). After plate conditioning, the mixed samples were loaded onto the SPE plate, and steroids were subsequently eluted in two steps: 1) twice with 500 µL dichlormethane into the same deep well plate and 2) with 600 µL acetonitrile into another deep well plate. After drying with nitrogen, the dichloromethane fraction was dissolved in 50 µL methanol/water (25/75 v/v), whereas the acetonitrile fraction was diluted with 400 µL water. Both plates were centrifuged at 50×*g* and placed into the cooled auto sampler (10 °C) for LC–MS/MS measurements. The LC-separation of both fractions was performed using 470 mL ultrapure water and the content of three ampules of the kit as mobile phase A and acetonitrile/methanol/ultrapure water v/v/v 85/10/5 as mobile phase B (initial 35%, rising up to 100%). After sample injection (20 µL), steroids were separated at a flow rate of 300 µL and at 45 °C column temperature on the HPLC column for the Absolute*IDQ*™ Stero17 Kit combined with the precolumn SecurityGuard Cartridge C18 4 × 2 mm (for HPLC, Phenomenex Cat No. AJ0-4286; Phenomenex, Aschaffenburg, Germany). The method of the Absolute*IDQ*™ Stero17 Kit were in conformance with the EMEA-Guideline^54^, which implies proof of reproducibility within a given error range. Analytical specifications for LOD, LLOQ and ULOQ (lower and upper limit of quantification), specificity, linearity, precision, accuracy, reproducibility, and stability were determined experimentally by Biocrates and are described in the user manual AS-STERO17-3.

Data evaluation for quantifying the steroid concentrations and quality assessment was performed with the software MultiQuant 3.0.1 (Sciex) and the Met*IDQ*™ software package, which is an integral part of the Absolute*IDQ*™ Stero17 Kit. Metabolite concentrations were calculated using internal standards and reported in nM or ng/mL. Due to unspecific contaminations, pregnanediol and pregnenolone could not be detected in 6 out of 38 samples at week 7 a.p. [2 NBCS, 4 HBCS], and 4 out of 38 samples after calving at week 1 [1 NBCS, 3 HBCS], week 3 [2 NBCS, 2 HBCS], and week 12 [4 HBCS].

### Quantification of steroid hormones in scAT

In bovine scAT, the same steroid hormones (except pregnenolone, pregnanediol, and aldosterone) as already described for serum were determined by LC–MS/MS based on the Absolute*IDQ*™ Stero17 assay (Biocrates Life Science AG). The QTRAP 5500 triple quadrupole system (Sciex) was equipped with a Turbo V ion spray interface and coupled to an Agilent 1290 Infinity UHPLC-system (two G7120A binary pumps, a G7116B column thermostat), and a G7167B multi-sampler (Agilent Technologies). Instrument control and data acquisition were performed with Analyst Software Version 1.7, and for data evaluation MultiQuant Version 3.0.3 was used (Sciex). Sample processing for steroid analysis in scAT was divided in two main steps. The first step was an in-house method (Helmholtz Zentrum München). The extracts obtained during this procedure were used to perform the second step of sample processing using the Absolute*IDQ*™ Stero17 assay (Biocrates Life Science AG). For steroid extraction, scAT (100 mg), internal standard solution (10 µL), methanol (240 µL), and pure water (250 µL) were homogenized (Precellys® 24, Bertin instruments, Montigny-le-Bretonneux, France). After homogenization, methanol (1 mL) was added, vortexed and incubated for 10 min at 50 °C and 800 rpm (Thermomix comfort, Eppendorf, Hamburg, Germany). Afterwards, tubes were centrifuged at 15,000×*g* and 4 °C, for 15 min (Mikro 200R Hettich centrifuge, Bäch, Switzerland) and subsequently stored at −20 °C for 30 min. The supernatant was transferred to a glass vial and evaporated to dryness (Barkey vapotherm, Barkey GmbH, Leopoldshöhe, Germany) under nitrogen at 30 °C. Finally, the residue was dissolved in methanol/water (250 µL; 5/95), and transferred to a 96 well plate provided with the Absolute*IDQ*™ Stero17 assay (Biocrates Life Science AG). In addition, blanks (250 µL), calibration solutions, and quality control samples were pipetted to the well plate. Finally, the steroids were subsequently eluted from the SPE plate. The first extract was obtained by elution with dichloromethane. After evaporation of the eluent, the residue was dissolved in methanol/water (50 µL; 25/75 vol/vol). This extract contained the steroids except for DHEA-S. A second extract was obtained by eluting the SPE-plate with acetonitrile. The eluate was diluted with water to get a final volume of 600 µL (acetonitrile/water 66/34 vol/vol) containing DHEA-S. Both extracts were subjected to LC–MS/MS analysis.

The liquid chromatographic separation of the steroids was performed on a core–shell column (Kinetex RP-18, 1.8 µm, 150 × 2.1 mm I.D. with a Security Guard Ultra Cartridge 2.1 mm I.D) from Phenomenex. During separation, the column was operated at 45 °C at a flow rate of 0.3 mL/min applying elution with binary gradients from 65% A to 0% A (A: formic acid (0.1%)/2-propanol, 97/3, v/v; B: acetonitrile/methanol/water, 85/10/15, v/v/v). The injection volume was 20 µl for the dichloromethane (steroids except for DHEA-S) and 10 µL for the acetonitrile extract (DHEA-S). Tandem mass spectrometric detection and quantification of the steroids were done by electrospray ionization and sMRM.

Quality parameters were assessed to demonstrate the performance of the analytical method for the determination of steroids in scAT. Regardless of the internal standards, recoveries of the steroids were between 19.9% and 101% with CVs from 6.9% to 39.6% (n = 10). Considering the internal standards, recoveries were between 48.9% and 111% with CVs from 5.5% to 19.0%. The calculation of recovery and repeatability based on the respective internal standards significantly improved the reliability and was thus recognized as indispensable; moreover, the use of substance-specific, individual internal standards is necessary. The LODs and LOQs were calculated based on signal-to-noise ratios 3:1 and 10:1, respectively. The LODs were between 0.002 ng/g and 0.3 ng/g scAT, LOQs ranged from 0.007 ng/g to 0.9 ng/g scAT. Imprecisions for within-day-analysis (n = 7)—as a measure of repeatability—ranged from 6.8% to 15.5% (CVs). The calibration functions were linear with regression coefficients > 0.995.

### Extraction of RNA and quantitative real-time PCR in scAT and liver samples

Details of the RNA extraction and cDNA synthesis were described previously^51^. After tissue homogenization with the Precellys 24 system (VWR/Peqlab Biotechnologie, Erlangen, Germany) total RNA was extracted from liver and scAT by using the TRIzol reagent (Invitrogen/Life Technologies, Carlsbad, CA, USA) according to the manufacturer´s protocol. The RNA was purified with spin columns according to the Qiagen kit protocol (RNeasy Mini Kit, Qiagen GmbH, Hilden, Germany). The concentration of total RNA and the purity was quantified at 260 nm and 280 nm using the Nanodrop 1000 (peQLab Biotechnology GmbH, Erlangen, Germany). For cDNA synthesis, a reverse transcription of 250 ng total RNA per 20 µL reaction volume was performed with RevertAid reverse transcriptase (Thermo Scientific GmbH, Dreieich, Germany) according to the manufacturer’s instructions with a Multicycler PTC 200 (MJ Research Inc, Watertown, MA, USA). Quantitative real-time PCR (qPCR) was carried out using an MX3000p PCR cycler (Stratagene, Amsterdam, the Netherlands, and Agilent, Santa Clara, CA, USA) in accordance with the Minimum Information for Publication of Quantitative Real-Time PCR Experiments (MIQE) guidelines^55^. The qPCR conditions and primer sequences used in the present study are presented in Table 1. Samples were run as triplicates in a total volume of 10 µL, with 2 µL of cDNA (diluted 1:4) as a template, 1 µL of assay-specific primer mix, 2 µL of water, and 5 µL of DyNAmo ColorFlash SYBR Green qPCR Kit (Thermo Fisher Scientific, Dreieich, Germany). Each run included a negative-template control for quantitative PCR, as well as a negative-template control and no-reverse-transcriptase control of cDNA. Relative quantification of the target genes, i.e. *HSD11B1*, *HSD3B1*, *HSD17B12*, *StAR*, and *CYP21* was performed with standard curves using cDNA serial dilutions to calculate the abundance based on run-specific PCR efficiency. For each PCR, a set of two inter-run calibrators was used to correct for inter-run variation.

**Table 1:** Primer characteristics of target and reference genes used in adipose tissue and liver and the real-time polymerase chain reaction conditions.

	Sequences (5′–3′)	NCBI Accession No	Fragment size (bp^3^)	Annealing (s/◦C)^4^	Extension (s)^5^
**Target Genes**^1^					
*HSD17B12*					
Forward	GCTGCTAAAACCCTGACCCA	NM_001101307.1	101	60/59	60
Reverse	GAGTGGCCTGGTGTCATTCA				
*CYP21*					
Forward	CGTGAAGGGCACTGAGAAAT	NM_174639.1	100	60/59	60
Reverse	AGGTGGGAGCTGAACGTCTA				
*StAR*					
Forward	GTGGATTTTGCCAATCACCT	NM_174189.2	202	30/62	15
Reverse	TTATTGAAAACGTGCCACCA				
*HSD3B1*					
Forward	TGTTGGTGGAGGAGAAGGATCTG	NM_174343	207	30/59	15
Reverse	GCATTCCTGACGTCAATGACAGAG				
*HSD11B1*					
Forward	AAGCAGACCAACGGGAGCATT	AF548027	112	60/60	60
Reverse	GGAGAAGAACCCATCCAGAGCA				
**Reference Genes**^2^					
*LRP10*					
Forward	CCAGAGGATGAGGACGATGT	BC149232	139	30/61	20
Reverse	ATAGGGTTGCTGTCCCTGTG				
*POL2*					
Forward	GAAGGGGGAGAGACAAACTG	X63564	86	60/60	30
Reverse	GGGAGGAAGAAGAAAAAGGG				
*HPCAL1*					
Forward	CCATCGACTTCAGGGAGTTC	NM_001098964	99	30/60	30
Reverse	CGTCGAGGTCATACATGCTG				
*EIF3K*					
Forward	CCAGGCCCACCAAGAAGAA	NM_001034489	125	45/59	30
Reverse	TTATACCTTCCAGGAGGTCCATGT				
*EMD*					
Forward	GCCCTCAGCTTCACTCTCAGA	NM_203361	100	45/59	30
Reverse	GAGGCGTTCCCGATCCTT				

^1^HSD17B12 = 17 ß-hydroxysteroid dehydrogenase type 12; CYP21 = steroid 21-hydroxylase; StAR = steroidogenic acute regulatory protein; HSD3B1 = 3β-Hydroxysteroid dehydrogenase type 1; HSD11B1 = 11ß-hydroxysteroid dehydrogenase type 1.

^2^LRP10 = lipoprotein receptor-related protein 10; POL2 = RNA polymerase II; HPCAL1 = hippocalcin-like protein 1; EIF3K = eukaryotic translation initiation factor 3 subunit K; EMD = Emerin.

^3^Base pairs.

^4^Initial denaturation for 10 min at 90 °C; denaturation for 30 s at 95 °C; 40 cycles, except for *StAR*, *HSD3B1*, and *HPCAL1* (35 cycles), *LRP10* (33 cycles).

^5^Extension at 72 °C.

Target genes were normalized based on the most stable reference genes in liver and scAT, i.e. low-density lipoprotein receptor-related protein 10 (*LRP10*), RNA polymerase II (*POL2*), eukaryotic translation initiation factor 3 subunit K (*EIF3K*), hippocalcin-like protein 1 (*HPCAL1*), and emerin (*EMD*), determined by geNorm^PLUS^ algorithms of qBASE^plus^ 3.1 software (Biogazelle, Ghent, Belgium).

### Statistical analysis

Statistical analyses for steroid hormones and steroidogenic enzymes were carried out by using a linear mixed model with repeated measurements (IBM SPSS Version 21). Before statistical analysis, the normality of data distribution was tested using the Shapiro–Wilk test using the UNIVARIATE procedure and evaluated visually by plotting residuals. All dependent data (steroids, steroidogenic enzymes mRNA) were not normally distributed; therefore, data were log (base 10) transformed to meet the assumptions of normality and homoscedasticity of the residuals. The data presented in this paper show the non-transformed values of the data (mean ± SEM); however, all P-values were calculated using the transformed data. The mixed models used herein contained the fixed effects of treatment (group: HBCS and NBCS), time (weeks relative to parturition), and the interaction between treatment and time, while the individual “cow” was considered as a random factor. The effect of BCS antepartum was included in the model as a fixed effect of the group (treatment). Moreover, parity (defined in classes) and the interaction with time was considered as a fixed effect; however, when insignificant it was excluded from the model. Multiparous cows were assigned to parity classes regarding their number of lactations at the beginning of the experimental period (i.e. BCS grouping): class 1: = 1st parity cows entering 2nd parity (n = 6), class 2: ≥ 2nd and < 4th parity (n = 14); class 3: ≥ 4th parity (n = 18). The Bonferroni correction was used for multiple comparisons. Differences with *P* ≤ 0.05 were considered significant and a trend was defined at 0.05 > *P* ≤ 0.10. Relationships between variables (non-transformed data) were tested by Spearman correlation using R^56^. Associations were tested for the periods before and after parturition, for the entire experimental period, as well as for each group separately. Correlation figures for the whole period were computed using the R package *corrplot*^57^. Correlations were shown based on their significance level of *P* < 0.05, whereas non-significant correlations were not illustrated (“blank”). Moreover, the web-based metabolomics data processing tool MetaboAnalyst 4.0 was used for principal component analysis (PCA) and heat map (distance measure using euclidean and clustering algorithm using ward) of the steroids data^58^. Before data analysis, a data integrity check was performed to make sure that all the necessary information had been collected. Variables containing more than 0.6% missing values (i.e. values lower than LOD) were not considered for steroid PCA. The metabolite data were transformed using the generalized log transformation and then Pareto-scaled (mean-centred and divided by the square root of the standard deviation of each variable) to correct for heteroscedasticity, to reduce the skewness of the data, and to reduce mask effects^59^.

### Supplementary Information

Supplementary Information 1.

Supplementary Information 2.

Supplementary Information 3.

Supplementary Information 4.

Supplementary Information 5.

Supplementary Information 6.

Supplementary Information 7.

## Data Availability

The data that support the findings of this study are available from the corresponding author, [S. Häussler], upon reasonable request.
