# Author Correction: Blood and adipose tissue steroid metabolomics and mRNA expression of steroidogenic enzymes in periparturient dairy cows differing in body condition

**DOI:** 10.1038/s41598-024-53305-8

**Published:** 2024-02-15

**Authors:** K. Schuh, S. Häussler, H. Sadri, C. Prehn, J. Lintelmann, J. Adamski, C. Koch, D. Frieten, M. H. Ghaffari, G. Dusel, H. Sauerwein

**Affiliations:** 1 Institute of Animal Science, Physiology Unit, University of Bonn, 53115 Bonn, Germany; 2 Department of Life Sciences and Engineering, Animal Nutrition and Hygiene Unit, University of Applied Sciences Bingen, 55411 Bingen am Rhein, Germany; 3 Department of Clinical Science, Faculty of Veterinary Medicine, University of Tabriz, 5166616471 Tabriz, Iran; 4 Helmholtz Zentrum München, German Research Center for Environmental Health, Metabolomics and Proteomics Core, 85764 Neuherberg, Germany; 5 Institute of Experimental Genetics, Helmholtz Zentrum München, German Research Center for Environmental Health, 85764 Neuherberg, Germany; 6 Department of Biochemistry, Yong Loo Lin School of Medicine, National University of Singapore, Singapore, 117597 Singapore; 7 Institute of Biochemistry, Faculty of Medicine, University of Ljubljana, 1000 Ljubljana, Slovenia; 8 Educational and Research Centre for Animal Husbandry, Hofgut Neumuehle, 67728 Muenchweiler an der Alsenz, Germany; 9 Thünen Institute of Organic Farming, 23847 Westerau, Germany

Correction to: *Scientific Reports* 10.1038/s41598-022-06014-z, published online 10 February 2022

The original version of this Article contained errors in Figure 3 where the panels (q) and (r) with the two hormones, estrone and estradiol, were omitted. The original Figure 3 and accompanying legend appear below.

**Figure 3:**
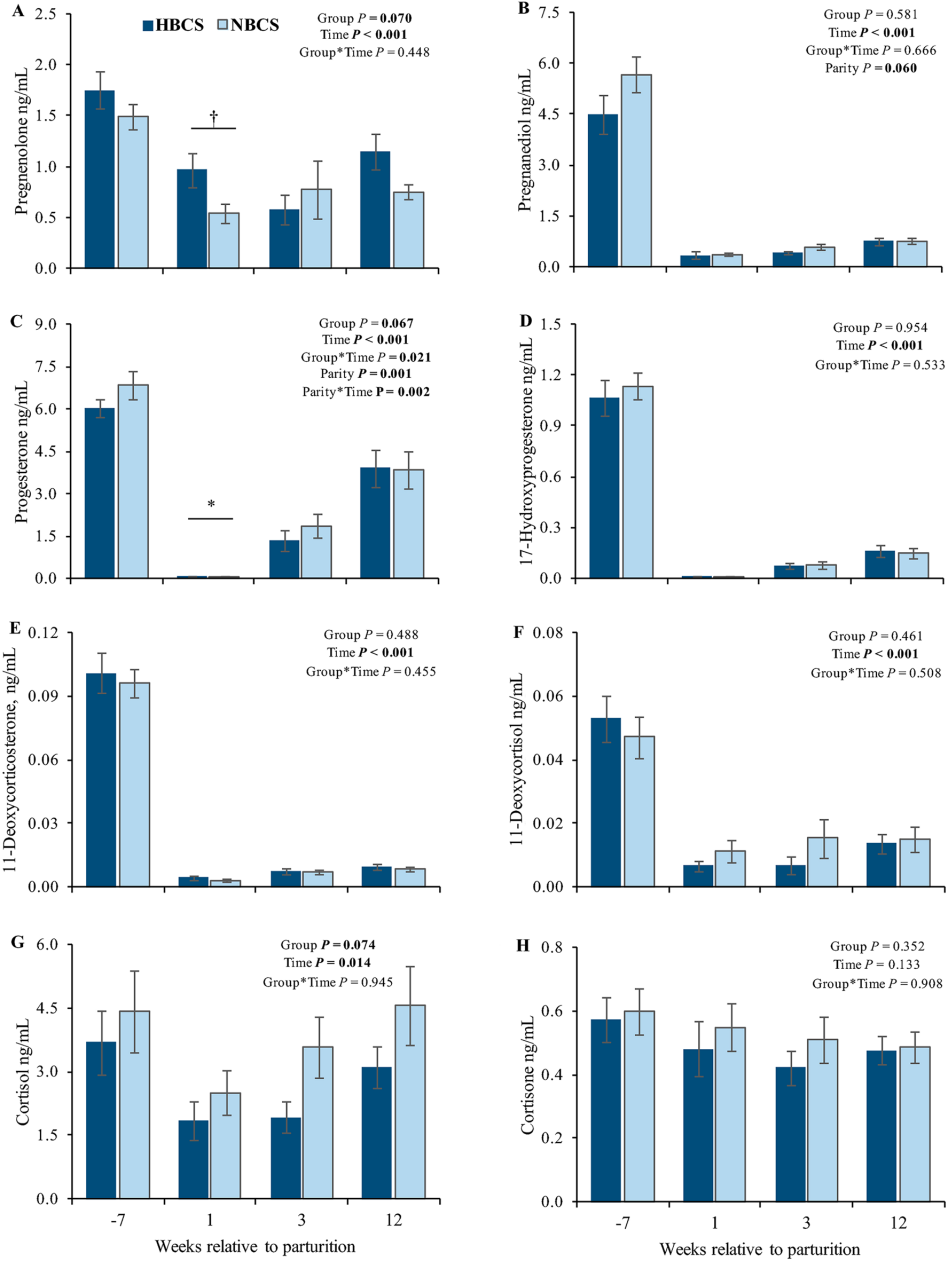

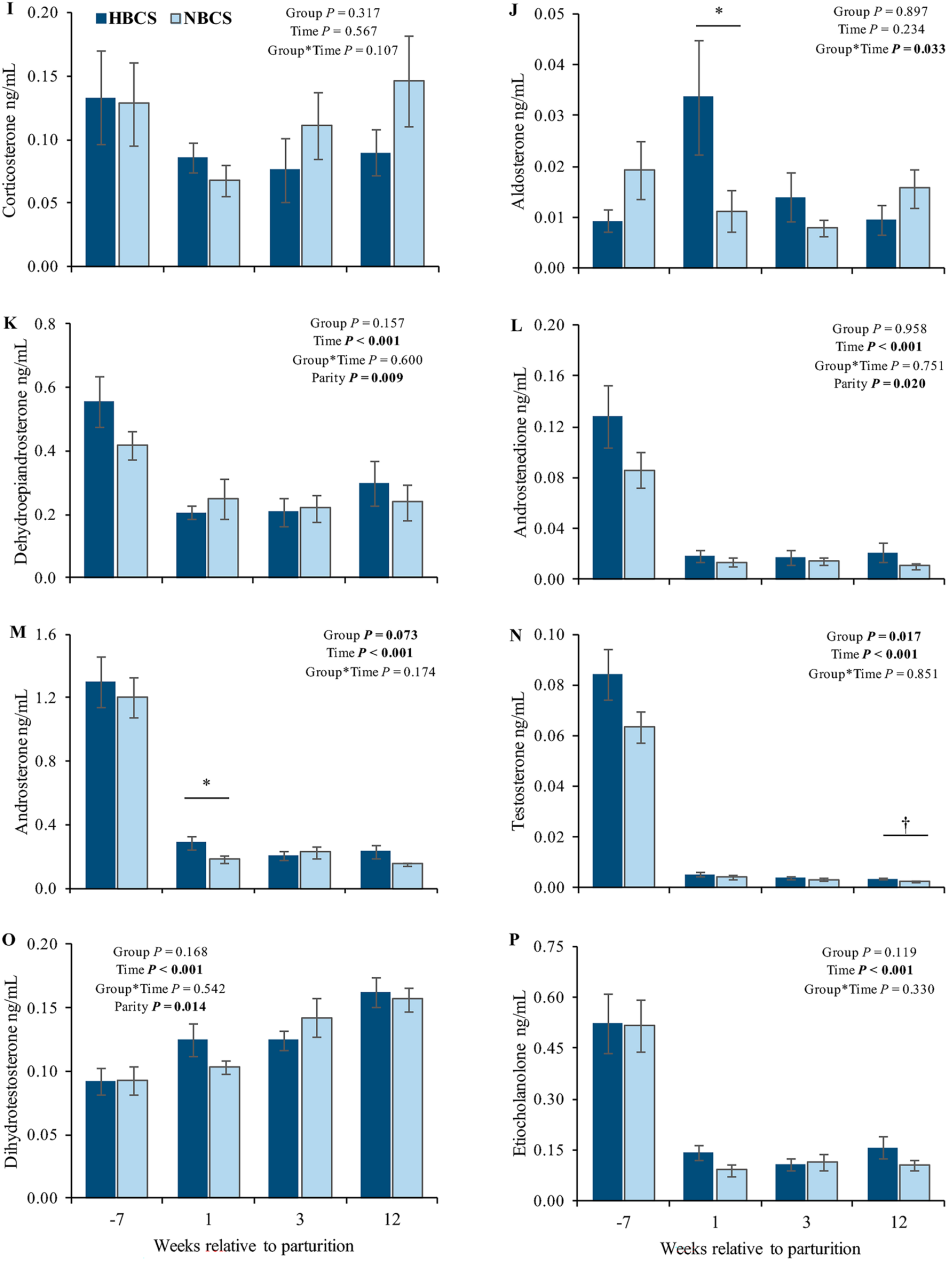
The caption to be typeset alongside it: Steroid hormones in serum. Changes of the steroid concentrations (ng/mL) in serum of cows with normal versus high body condition score (NBCS, HBCS) at week 7 ante partum, as well as week 1, 3 and 12 postpartum. Data are given as means ± SEM. Asterisks (*) indicate differences (*P* ≤ 0.05) between HBCS and NBCS cows within the time points. Trends (*P* ≤ 0.10) for differences between the groups are indicated by daggers (†).

The original Article has been corrected.

