# Supplementary material for: Blood and adipose tissue steroid metabolomics and mRNA expression of steroidogenic enzymes in periparturient dairy cows differing in body condition
Source: Sci Rep. 2022 Feb 10;12:2297. doi: 10.1038/s41598-022-06014-z (PMC8831572; doi:10.1038/s41598-022-06014-z)
Supplement: Supplementary file 1 — Supplementary Information 1. [file 41598_2022_6014_MOESM1_ESM.docx]

**Supplemental Figure 1.** Changes in body condition score (BCS) and backfat thickness (BFT) from week 7 ante partum to week 13 postpartum of cows with normal versus high body condition score (NBCS, HBCS; each n = 19). Data are given as means ± SEM. Asterisks indicate differences (*: *P* ≤ 0.05; **: *P* ≤ 0.01; ***: *P* ≤ 0.001; #: 0.05 > *P* ≤ 0.10) between HBCS and NBCS within one time point. The vertical dashed line indicates parturition. The vertical dashed line indicates parturition. Data were already published by Schuh et al. (2019).
