# Supplementary material for: Blood and adipose tissue steroid metabolomics and mRNA expression of steroidogenic enzymes in periparturient dairy cows differing in body condition
Source: Sci Rep. 2022 Feb 10;12:2297. doi: 10.1038/s41598-022-06014-z (PMC8831572; doi:10.1038/s41598-022-06014-z)
Supplement: Supplementary file 2 — Supplementary Information 2. [file 41598_2022_6014_MOESM2_ESM.docx]

**Supplemental Figure 2.** Changes in non-esterified fatty acids (nmol/L) and β-hydroxybutyrate (nmol/L) from week 7 ante partum to week 13 postpartum of cows with normal versus high body condition score (NBCS, HBCS; each n = 19). Data are given as means ± SEM. Asterisks indicate differences (*: *P* ≤ 0.05; **: *P* ≤ 0.01; ***: *P* ≤ 0.001; #: 0.05 > *P* ≤ 0.10) between HBCS and NBCS within one time point. The vertical dashed line indicates parturition. Data were already published by Schuh et al. (2019).
