# Supplementary material for: Blood and adipose tissue steroid metabolomics and mRNA expression of steroidogenic enzymes in periparturient dairy cows differing in body condition
Source: Sci Rep. 2022 Feb 10;12:2297. doi: 10.1038/s41598-022-06014-z (PMC8831572; doi:10.1038/s41598-022-06014-z)
Supplement: Supplementary file 3 — Supplementary Information 3. [file 41598_2022_6014_MOESM3_ESM.docx]

**Supplemental Figure 3.** Yields of (A) milk, (B) energy-corrected milk, (C) milk fat and (D) milk protein, and concentrations of (E) lactose and (F) urea in milk in high body condition score (HBCS) or normal body condition score (NBCS) cows from 1 to 14 weeks *postpartum* (time = weeks relative to calving). Results are presented as means ± SEM. Significant differences between the groups are indicated with asterisks (*) when P ≤ 0.05 or (**) when P ≤ 0.01 at a given time point, respectively. Trends (P ≤ 0.10) for differences between the groups at a given time point are indicated by (#). Data were already published by Schuh et al. (2019).
