# Supplementary material for: Blood and adipose tissue steroid metabolomics and mRNA expression of steroidogenic enzymes in periparturient dairy cows differing in body condition
Source: Sci Rep. 2022 Feb 10;12:2297. doi: 10.1038/s41598-022-06014-z (PMC8831572; doi:10.1038/s41598-022-06014-z)
Supplement: Supplementary file 4 — Supplementary Information 4. [file 41598_2022_6014_MOESM4_ESM.docx]

**Supplemental Table 1.** Ingredients and chemical composition of the diets (from Schuh et al., 2019)

| Item | Late lactation | |  | Dry period |  | Early lactation |
| --- | --- | --- | --- | --- | --- | --- |
|  | Wk 15 to 7 a.p. | |  | Wk 7 a.p. to parturition |  | Wk 1 to 14 p.p. |
|  | HBCS | NBCS |  | HBCS / NBCS |  | HBCS / NBCS |
| Ingredient |  |  |  |  |  |  |
| Grass silage | 22.4 | 32.0 |  | 32.0 |  | 22.4 |
| Corn silage | 20.7 | 32.0 |  | 32.0 |  | 20.7 |
| Pressed beet pulp silage | 12.5 | - |  | - |  | 12.5 |
| Hay | 5.5 | 5.4 |  | 5.4 |  | 5.5 |
| Straw | 2.3 | 4.1 |  | 4.1 |  | 2.3 |
| Vitamin and mineral mix^1^ | 0.4 | 0.7 |  | 0.7 |  | 0.4 |
| Concentrate^2^ | 36.2 | 25.8 |  | 25.8 |  | 36.2 |
| Chemical composition |  |  |  |  |  |  |
| ME (MJ/kg DM) | 10.8 | 10.6 |  | 10.6 |  | 10.8 |
| NE_L_ (MJ/kg DM) | 7.2 | 6.8 |  | 6.8 |  | 7.2 |
| Crude protein (g/kg DM) | 170 | 157 |  | 157 |  | 170 |
| Utilizable crude protein (g/kg DM) | 156 | 149 |  | 149 |  | 156 |
| aNDF_OM_ ^†^ (g/kg DM) | 359 | 382 |  | 382 |  | 359 |
| ADF_OM_ ^‡^ (g/kg DM) | 204 | 223 |  | 223 |  | 204 |
| Ruminal N balance (g/d) | 3.4 | 2.3 |  | 2.3 |  | 3.4 |

^1^Provided per kg total mixed ration (on DM basis): Ca, 0.36 g; P, 0.36 g; sodium, 0.36 g; Mg, 0.40 g; Zn, 28 mg; Mn, 17 mg; Cu, 6.0 mg; Co, 0.24 mg; I, 0.80 mg; Se, 0.21 mg; vitamin A, 4.000 IU, vitamin D, 600 IU, vitamin E, 20 mg (RINDAMIN K11 ATG, Schaumann, Pinneberg, Germany).

^2^Concentrate portion consisting of barley (6.5% of DM), corn grain (8.8% of DM), soybean meal (5.9% of DM), and canola meal (6.5% of DM)

^†^ aNDF_OM_, ash free neutral detergent fiber of organic matter

^‡^ ADF_OM_, acid detergent fiber of organic matter
