# Supplementary material for: Blood and adipose tissue steroid metabolomics and mRNA expression of steroidogenic enzymes in periparturient dairy cows differing in body condition
Source: Sci Rep. 2022 Feb 10;12:2297. doi: 10.1038/s41598-022-06014-z (PMC8831572; doi:10.1038/s41598-022-06014-z)
Supplement: Supplementary file 5 — Supplementary Information 5. [file 41598_2022_6014_MOESM5_ESM.docx]

**Supplemental Table 2:** Body condition score (BCS), back fat thickness (BFT, cm), and body weight (BW, kg) loss of animals with high (HBCS; n = 19) versus normal (NBCS; n = 19) BCS before calving. Absolute losses are given as mean ± SEM, relative variations (%) are presented in parenthesis. P-values represent differences between groups within one time slot.

| **Time** |  | **wk -1 until +3** | **wk -1 until +7** | **wk -1 until +11** | **wk -1 until +15** |
| --- | --- | --- | --- | --- | --- |
| **Group** | **Parameter** |  |  |  |  |
| **HBCS** | BCS loss | 0.62 ± 0.06 (15 %) | 0.97 ± 0.06 (23 %) | 1.08 ± 0.06 (26%) | 1.17 ± 0.09 (28 %) |
|  | BFT loss, cm | 0.52 ± 0.07 (21 %) | 1.05 ± 0.10 (43 %) | 1.28 ± 0.11 (53%) | 1.37 ± 0.14 (64 %) |
|  | BW loss, kg | 140 ± 7 (17 %) | 171 ± 13 (20 %) | 177 ± 13 (21 %) | 215 ± 13 (25 %) |
|  |  |  |  |  |  |
| **NBCS** | BCS loss | 0.42 ± 0.07 (12 %) | 0.63 ± 0.06 (18 %) | 0.68 ± 0.08 (19%) | 0.82 ± 0.11 (23 %) |
|  | BFT loss, cm | 0.30 ± 0.06 (23 %) | 0.51 ± 0.06 (38 %) | 0.66 ± 0.06 (49%) | 0.69 ± 0.08 (51%) |
|  | BW loss, kg | 102 ± 7 (14 %) | 109 ± 8 (15 %) | 112 ± 8 (15 %) | 121 ± 10 (16 %) |
| *P* value*^1^* | BCS | 0.036 | < 0.001 | < 0.001 | 0.020 |
|  | BFT | < 0.001 | < 0.001 | < 0.001 | < 0.001 |
|  | BW | 0.001 | < 0.001 | < 0.001 | < 0.001 |

^1^*P*-value based on UNIANOVA (GLM)
