# Supplementary material for: Blood and adipose tissue steroid metabolomics and mRNA expression of steroidogenic enzymes in periparturient dairy cows differing in body condition
Source: Sci Rep. 2022 Feb 10;12:2297. doi: 10.1038/s41598-022-06014-z (PMC8831572; doi:10.1038/s41598-022-06014-z)
Supplement: Supplementary file 6 — Supplementary Information 6. [file 41598_2022_6014_MOESM6_ESM.docx]

**Supplemental Table 3.** Number of cows and number of cases affected by clinical conditions occurring from calving to the first 6 weeks after calving in HBCS and NBCS cows (from Webb et al., 2020).

| Clinical condition | HBCS (n = 19) | | NBCS (n = 19) | |
| --- | --- | --- | --- | --- |
|  | No. cows | No. cases | No. cows | No. cases |
| Mastitis | 8 | 16 | 6 | 11 |
| Ketosis | 4 | 5 | 2 | 2 |
| Milk fever | 4 | 5 | 2 | 4 |
| Locomotion | 7 | 8 | 3 | 4 |
| Retained fetal membranes/endometritis | 1 | 1 | 2 | 2 |
| Other conditions^1^ | 3 | 4 | 3 | 3 |
| Total clinical conditions | 27 | 39 | 15 | 26 |

^1^ dystocia, inflammation of the conjunctiva of the eye
