# Supplementary material for: Blood and adipose tissue steroid metabolomics and mRNA expression of steroidogenic enzymes in periparturient dairy cows differing in body condition
Source: Sci Rep. 2022 Feb 10;12:2297. doi: 10.1038/s41598-022-06014-z (PMC8831572; doi:10.1038/s41598-022-06014-z)
Supplement: Supplementary file 7 — Supplementary Information 7. [file 41598_2022_6014_MOESM7_ESM.docx]

**Supplemental Table 4.** The steroids determined by the LC-MS/MS methods, their transitions, and the internal standards used for quantification. The first transition was used for quantification, the second one (if available) for qualification. Steroids are listed in the order of their elution from the analytical columns.

| Steroid | Transitions (m/z) | Internal standard |
| --- | --- | --- |
| Dehydroepiandrosterone  sulfate ^a^ | 367.2 → 97.0 367.2 → 80.1 | d6- Dehydroepiandrosterone  sulfate |
| Aldosterone ^b^ | 361.1 → 343.2 361.1 → 315.2 | d7-Aldosterone |
| Cortisol | 363.1 → 345.1 363.1 → 121.1 | d4-Cortisol |
| Cortisone | 361.1 → 163.1 361.1 → 105.0 | d7-Cortisone |
| Corticosterone | 347.1 → 329.2  347.1 → 121.1 | d8-Corticosterone |
| 11-Deoxycortisol | 347.1 → 97.1 347.1 → 109.1 | d5-11-Deoxycortisol |
| Estradiol | 255.1 → 159.1 255.1 → 133.2 | d3-Estradiol |
| Testosterone | 289.1 → 97.0 289.1 → 109.1 | d5-Testosterone |
| 11-Deoxycorticosterone | 331.1 → 97.1 331.1 → 109.1 | d8-11-Deoxycorticosterone |
| Estrone | 271.1 → 133.1 271.1 → 253.2 | d4-Estrone |
| Androstenedione | 287.1 → 97.1 287.1 →109.1 | d3-Androstenedione |
| Dehydroepiandrosterone | 271.1 → 253.2 271.1 → 133.1 | d4- Dehydroepiandrosterone |
| 17-Hydroxyprogesterone | 331.1 → 97.1 331.1 → 109.1 | d8-17-Hydroxyprogesterone |
| Dihydrotestosterone | 291.1 → 255.2 | d3-Diyhydrotestosterone |
| Etiocholanolone | 273.1 → 255.2 291.2 → 255.2 | d4-Etiocholanolone |
| Androsterone | 273.1 → 255.2 291.2 → 255.2 | d4-Androsterone |
| Progesterone | 315.1 → 109.1 315.1 → 97.1 | d9-Progesterone |

^a^: For negative ion mode (unscheduled MRM) the dwell time was 25 ms.

^b^: For positive ion mode (scheduled MRM) the target scan time per sMRM experiment was 0.5 s. Aldosterone was not determined in
 scAT.
